# Tectonic DSAEK for the Management of Impending Corneal Perforation

**DOI:** 10.1155/2012/916528

**Published:** 2012-12-05

**Authors:** Enrique O. Graue-Hernandez, Isaac Zuñiga-Gonzalez, Julio C. Hernandez-Camarena, Martha Jaimes, Patricia Chirinos-Saldaña, Alejandro Navas, Arturo Ramirez-Miranda

**Affiliations:** Department of Cornea and Refractive Surgery, Instituto de Oftalmologia “Conde de Valenciana”, Chimalpopoca 14, 06800 Mexico City, DF, Mexico

## Abstract

*Purpose*. To report a case of severe corneal thinning secondary to dry eye treated with a tectonic Descemet stripping automated lamellar keratoplasty (DSAEK) and amniotic membrane graft. *Methods*. A 72-year-old man with a history of long standing diabetes mellitus type 2 and dry eye presented with 80% corneal thinning and edema on the right eye and no signs of infectious disease, initially managed with topical unpreserved lubrication and 20% autologous serum drops. Eight weeks after, the defect advanced in size and depth until Descemetocele was formed. Thereafter, he underwent DSAEK for tectonic purposes. One month after the procedure, the posterior lamellar graft was well adhered but a 4 mm epithelial defect was still present. A multilayered amniotic membrane graft was then performed. *Results*. Ocular surface healed quickly and reepithelization occurred over a 2-week period. Eight months after, the ocular surface remained stable and structurally adequate. *Conclusion*. Tectonic DSAEK in conjunction with multilayered amniotic graft may not only provide structural support and avoid corneal perforation, but may also promote reepithelization and ocular surface healing and decrease concomitant inflammation.

## 1. Introduction 

Corneal perforations are a common complication of various corneal pathologies and can result in severe visual disability. Generally, they can be classified into traumatic and nontraumatic etiologies (most commonly secondary to infection or inflammation) [1]. Nontraumatic etiologies encompasses all causes of corneal perforations, including complications of infectious disease, neurotrophic ulcers, exposure keratitis, and keratitis sicca, being the later one of the most frequent causes [2, 3]. Management depends on the cause, size, severity, and location of the perforation. Current therapeutic strategies includes the placement of cyanoacrylate glue [4] with or without plastic drape plug [5], amniotic membrane [6], conjunctival flaps, and anterior lamellar [7] or penetrating keratoplasty. We report a case of impending corneal perforation due to dry eye and diabetic epitheliopathy successfully managed with Descemet stripping endothelial keratoplasty (DSAEK) and secondary placement of amniotic membrane multilayer graft. 

## 2. Case Report

A 72-year-old male with a history of long standing diabetes mellitus type 2 and dry eye presented to the Cornea Department with a 2-month history of decreased visual acuity and mild discomfort of the right eye. Upon examination, visual acuity was 20/80 OD and 20/40 OS. Biomicroscopy revealed adequate eyelid closure in both eyes with meibomian gland dysfunction. Tear meniscus was less than 1 mm and tear break-up time less than 7 seconds. The right cornea demonstrated superficial punctate keratitis, stromal edema, and a midperipheral 2 mm wide epithelial defect and corneal thinning of 80% with no signs of infectious disease. The anterior chamber had no signs of inflammation and a posterior chamber intraocular lens was placed in the capsular bag. Corneal aesthesiometry was marginally decreased in OD. The left cornea had mild inferior punctate keratitis and mild nuclear sclerosis in lens. Schirmer II test showed 5 mm and 12 mm OD, and OS respectively. The rest of the ophthalmologic exam was within normal limits. Initial workup included superficial scrapings for PCR for HSV-1, HSV-2, and VZV; also Rheumatoid Factor, antinuclear antibodies, anti-SSA, anti-SSB, anti-CCP, anti Hepatitis C, p-ANCA, and c-ANCA. All results were negative or within normal limits. He was initially managed with topical unpreserved lubricant (Lagricel Sodium Hyaluronate 0.4%, Sophia laboratories, Guadalajara, Mexico) and 20% autologous serum drops QID. The epithelial defect healed adequately and vision improved to 20/60. Eight weeks later, the defect increased in size to 4.7 × 4.0 mm and 90% depth—to reach a Descemetocele—and vision decreased to 20/400. After the informed consent and discussion of possible complications he underwent DSAEK surgery for tectonic purposes. 

Briefly, a donor endothelial lenticule of 72-year-old cornea with endothelial cell density of 2700 cells/mm^2^ was prepared using the Moria LSK microkeratome (Moria/Microtek, Inc., Doylestown, Pennsylvania) with a 350 microns head. Immediately after that the graft was trephined with an 8.5 mm punch. The recipient was prepared with a 5 mm superior scleral tunnel incision and an anterior chamber maintainer was placed nasally. Endothelial scraping and scoring were done being careful not to perforate the already fragile cornea. The lenticule was inserted using the Busin Glide (Moria, USA) and forceps. The main wound was sutured tightly and the anterior chamber was completely filled with air for a period of 10 minutes after which a 50% residual air bubble was left. 

**Figure 1:**
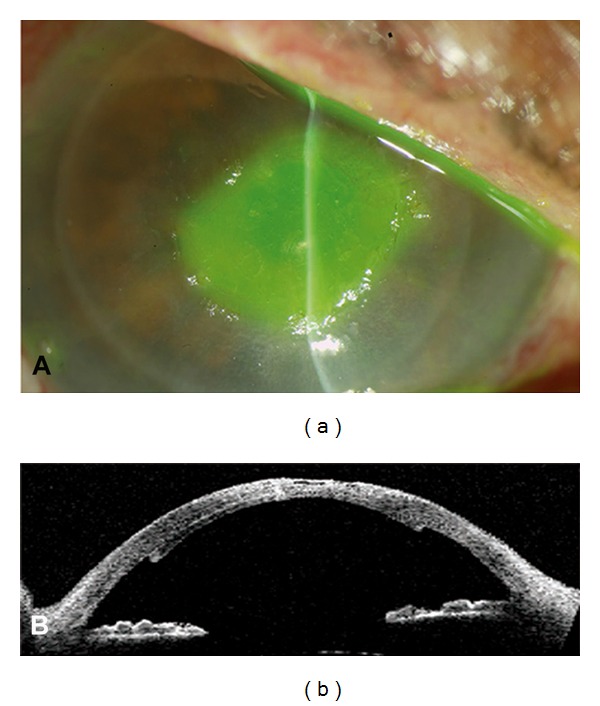
Day 1 postoperatively. (a) Slit lamp photography showing epithelial defect staining with fluorescein, mild corneal edema, and well-attached posterior lenticule. (b) Visante OCT showing epithelial defect, stromal thinning, and attachment of the posterior lenticule.

At postoperative day 1 the lenticule was attached, the anterior chamber was formed, and intraocular pressure was normal (Figure 1). Eye patch was placed and moxifloxacin 1% (Vigamoxi, Alcon laboratories, Fort Worth Texas, EU) and prednisolone acetate 1% (Prednefrin, Allergan, Los Angeles, CA, USA) were instilled QID. Lubrication with unpreserved Sodium Hyaluronate (Lagricel Sophia laboratories, Guadalajara, Mexico) was continued every hour. Pressure patch was placed and the patient was examined in the clinic every 72 hours for the next 14 days. 

**Figure 2:**
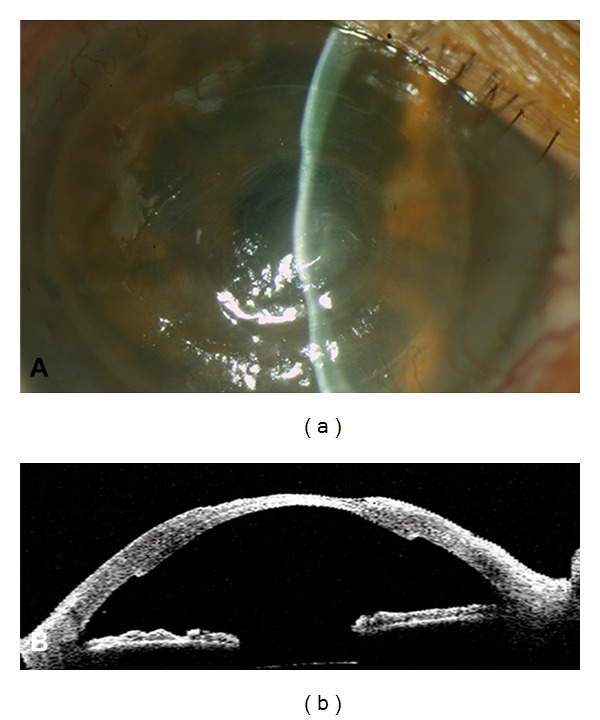
Month 1 postoperatively. (a) Slit lamp photography showing an integrated amniotic membrane graft, stromal thinning and adhered endothelial graft. (b) Visante OCT showing integrated amniotic membrane graft, stromal thinning, and well-attached endothelial graft.

At 1 month postoperatively the graft was still well adhered, but a 4.00 mm persistent epithelial defect was present with no signs of epithelial healing. A multilayer amniotic membrane graft using cryopreserved amniotic membrane (AMNIOCV; Instituto de Oftalmologia “Conde de Valenciana” IAP, Mexico City, Mexico) was then performed and sutured with 10-0 nylon. The ocular surface healed quickly and an epithelial healing occurred over a 2-week period (Figure 2). The sutures were removed and the topical medications reduced to unpreserved sodium hyaluronate (Lagricel Sophia) five to six times a day and topical and prednisolone acetate 1% (Allergan) QID and tapered over the next 4 months.

**Figure 3:**
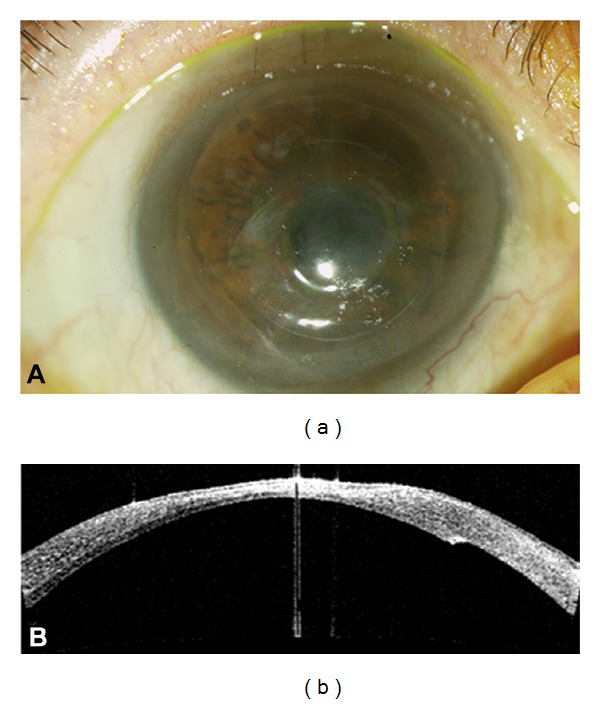
Month 8 postoperatively. (a) Slit lamp photography showing smooth and stable ocular surface, no epithelial defect, and mild stromal thinning. (b) Visante OCT showing a totally adhered posterior lenticule, a structurally stable cornea with a mild stromal thinning.

Eight months after the procedure the patient had a stable and healthy ocular surface with adequate corneal integrity (Figure 3). Penetrating keratoplasty to restore optical properties of the cornea and to promote visual rehabilitation is considered for the near future.

## 3. Discussion

Regardless of the etiology, when nontraumatic corneal thinning and perforations occur, it is considered an ophthalmic emergency and prompt treatment is needed to restore the anatomic and structural integrity of the eye. Tissue adhesives have been reported to achieve success up to 86%, but only in lesions smaller than 1 mm^2^ in size [8]. Lamellar anterior keratoplasty procedures have been used for perforations larger than 2 mm^2^ and whenever possible are preferred over penetrating procedures because of the reduction of the endothelial rejection, particularly in inflammatory disorders [9–11]. This procedure involves the use of sutures to attach the graft to the receptor tissue, and the presence of sutures has been associated with postoperative complications, such as microbial keratitis, corneal inflammatory infiltrates, and vascularization [12, 13]. All of these may ultimately affect the already compromised ocular surface by promoting inflammation, delaying the healing process of the entire ocular surface, and jeopardizing the success of the graft as structural support. The use of tissue adhesive to attach the graft instead of sutures in lamellar anterior keratoplasty may overcome this issue if a stable smooth interface is achieved [13]. 

The extraordinary success of orthotopic corneal allografts is in part attributed to the recognition of donor graft antigens by corneal antigen presenting cells (APCs) expressing MHC class II, which promote the anterior chamber-associated immune deviation and enhance graft survival by inducing immune tolerance [14, 15]. 

Amniotic membrane consists of an epithelial monolayer, a basement membrane, and an avascular stroma [16]. It has been reported that the amniotic membrane promotes epithelial migration, provides a scaffold for corneal repair, and has anti-inflammatory properties and antiproteinase activity, making it an ideal patching tool in corneal nontraumatic perforations, particularly in those of inflammatory origin [17, 18]. 

The success rate using multilayered methods leading to reepithelization in cases of corneal perforation or Descemetocele has been reported to be 73–80% [18]. Thus, we propose a novel “sandwich” technique for corneal perforations larger than 2 mm in which structural support is given by the posterior tectonic lenticule and the epithelial patch and anti-inflammatory treatment is achieved with the amniotic membrane graft.

Donor endothelial lenticules prepared with the use of a microkeratome allow for a section of corneal stroma ranging from 100 *μ*m to 200 *μ*m of thickness depending on the cutting technique used along with its Descemet membrane and endothelium [19]. When attached to the posterior corneal stroma, this lamella is able to restore the structural and physiological integrity of the cornea without a further modification of the ocular surface with sutures nor inducing more inflammation stimulus, as donor antigens may be harder to reach by the recipient APCs. If epithelial healing is still compromised, an amniotic membrane graft may address its repair and could reduce inflammation.

Posterior lamellar transplantation as a therapeutic strategy for corneal edema has evolved dramatically over the last decade with an increasing success in survival and improving visual results [20]. Although immunologic graft rejection is still a concern in DSAEK, rejection rates are comparable or even lower than in penetrating procedures [21, 22]. 

The restoration of the structural integrity of the eye is often difficult to achieve, and the use of posterior transplantation techniques, in the scenario of corneal perforations, may be advantageous over traditional penetrating or anterior lamellar procedures. Amniotic membrane grafting in conjunction with tectonic DSAEK (“sandwich” technique) may not only help to improve the structural aspect but may also promote epithelization and ocular surface restoration.
